# Supplementary material for: FLT3-ITD Measurable Residual Disease Monitoring in Acute Myeloid Leukemia Using Next-Generation Sequencing
Source: Cancers (Basel). 2022 Dec 12;14(24):6121. doi: 10.3390/cancers14246121 (PMC9776673; doi:10.3390/cancers14246121)
Supplement: Supplementary file 1 [file cancers-14-06121-s001.zip › cancers-2011631-supplementary/Cancers Supplementary_Figure-revised.pdf]

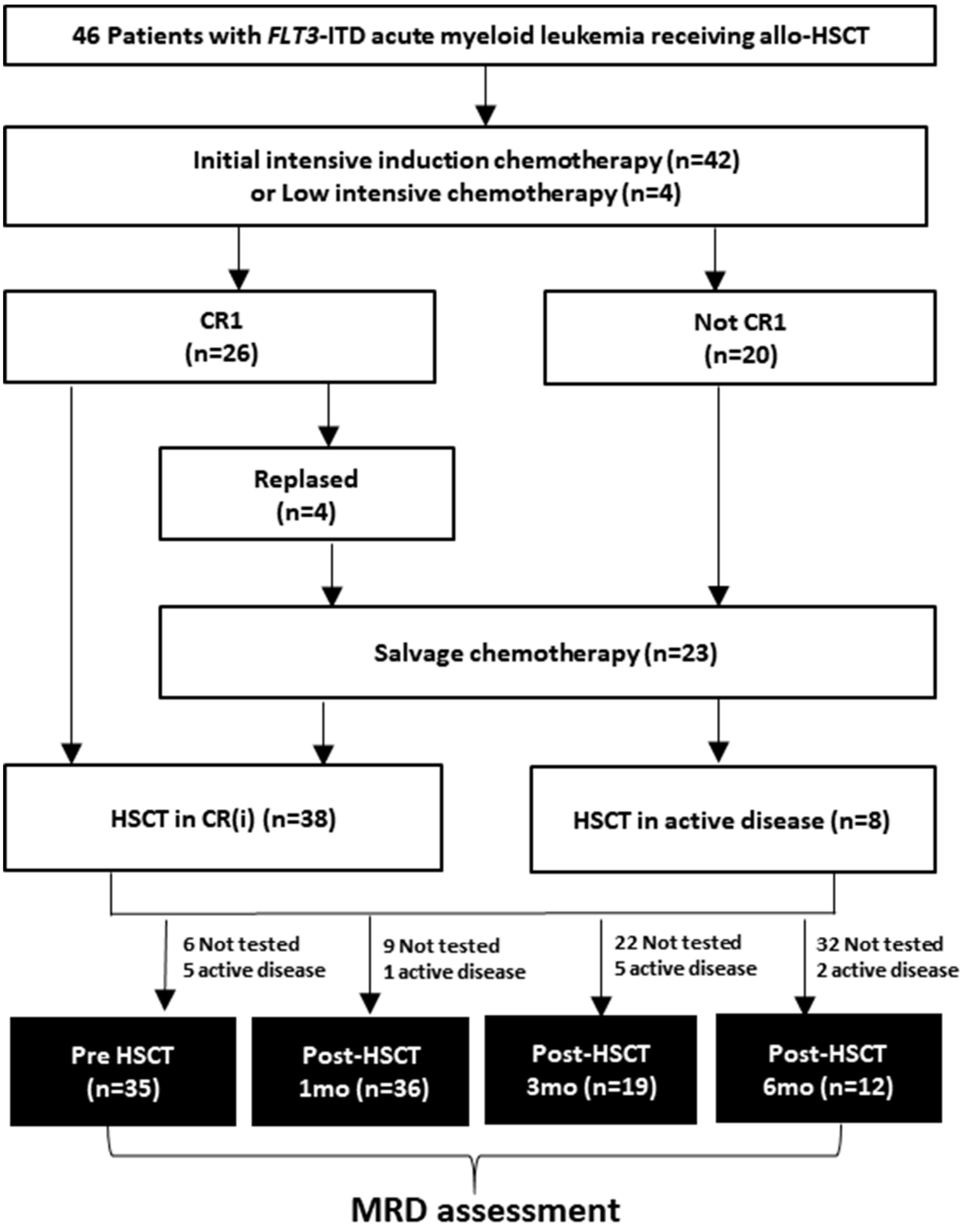

Figure S1. Treatment profile of the patients and selection for MRD assessments

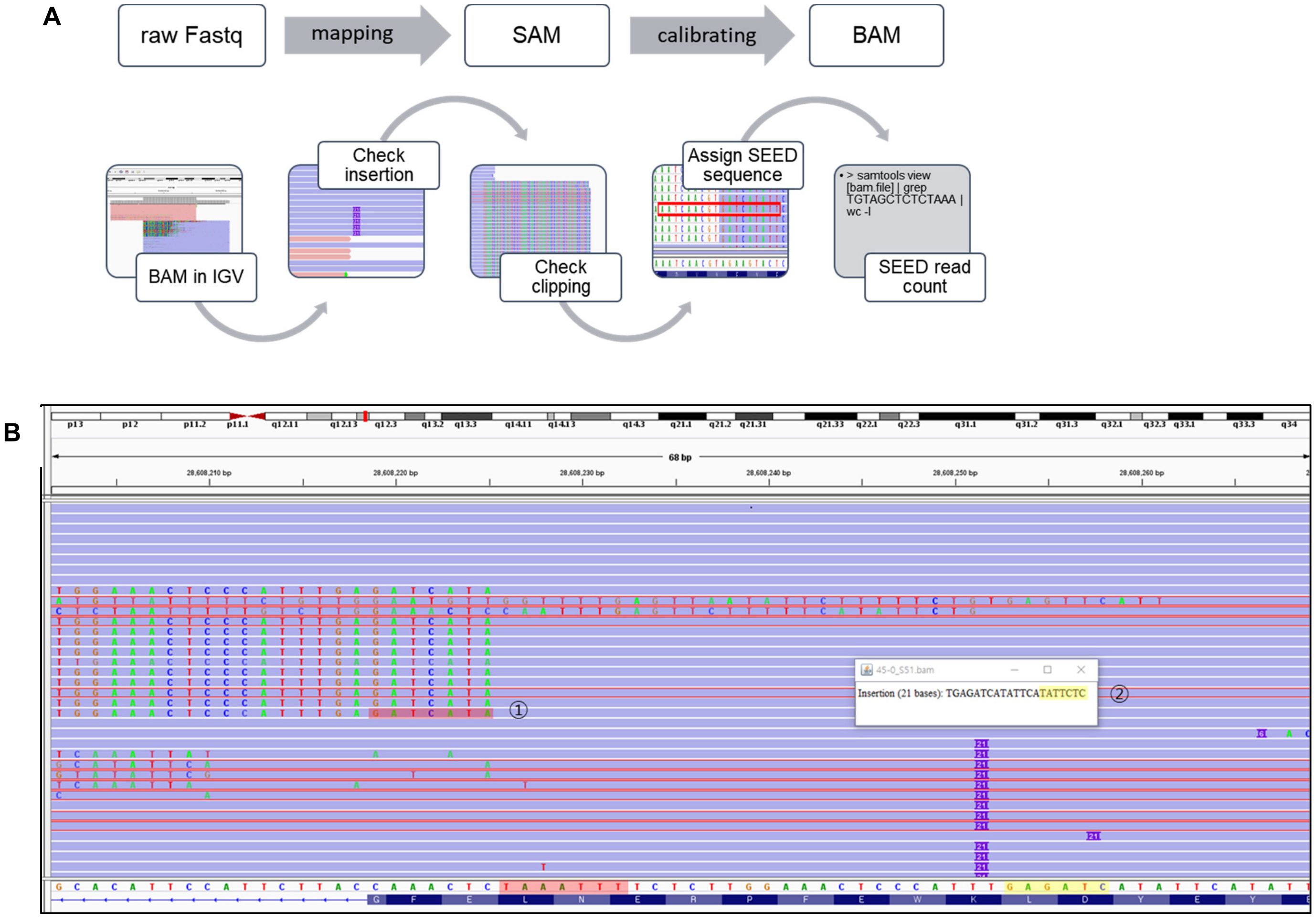

Figure S2. Procedure of the NGS-SEED algorithm and examplary assignment of SEED sequence.

C

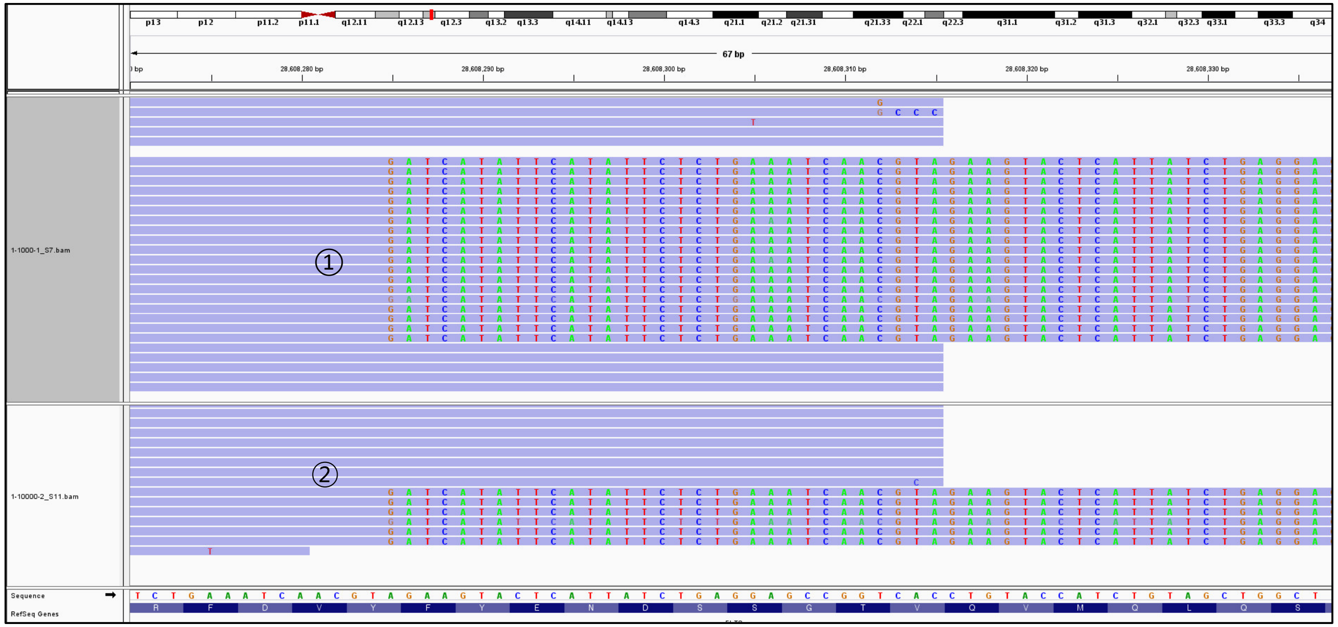

A: To identify SEED sequences, binary alignment map (BAM) files were visualized in Integrative Genomics View (IGV) software. Insertions or soft-clipped bases present in three or more of more than 10,000 reads were considered to be ITDs. VAF values were calculated as proportions of SEED-containing to total reads. The SAMtools script to count SEED reads from BAM files was:

> *> samtools view [bam.file] | grep GAAATCAACGGGAAACTCC | wc –l*

B: Example assignment of SEED sequence.

① Soft-clipped bases showing junctions of insertion and its origin. The SEED sequence was assigned as GATCATATAAATTT (red shading).

② ITD insertion visualized in IGV. The SEED sequence was assigned as TATTCTCGAGATC (yellow shading).

C: *FLT3*-ITD inspections for SEED assignment in:

① Sample diluted 10^{-3}.

② Sample diluted 10^{-4}.

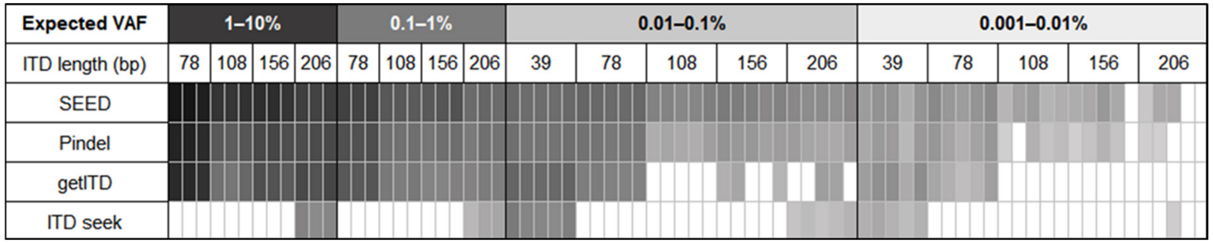

For 84 diluted clinical samples containing 5 different types of ITDs. Detection was 95.9% (71/74) by SEED, 93.2% (69/74) by Pindel, 66.2% (49/74) by getITD, and 29.7% (22/74) by ITDseek. The color scale indicates the measured VAF levels.

Figure S3. Detection capacities of bioinformatics tools in diluted clinical samples.

R^2 values are 0.909 for 39 bp (A); 0.988 for 78 bp (B); 0.981 for 108 bp (C); 0.868 for 156 bp (D); and 0.914 for 206 bp ITDs (E). Regression lines are shown in blue.

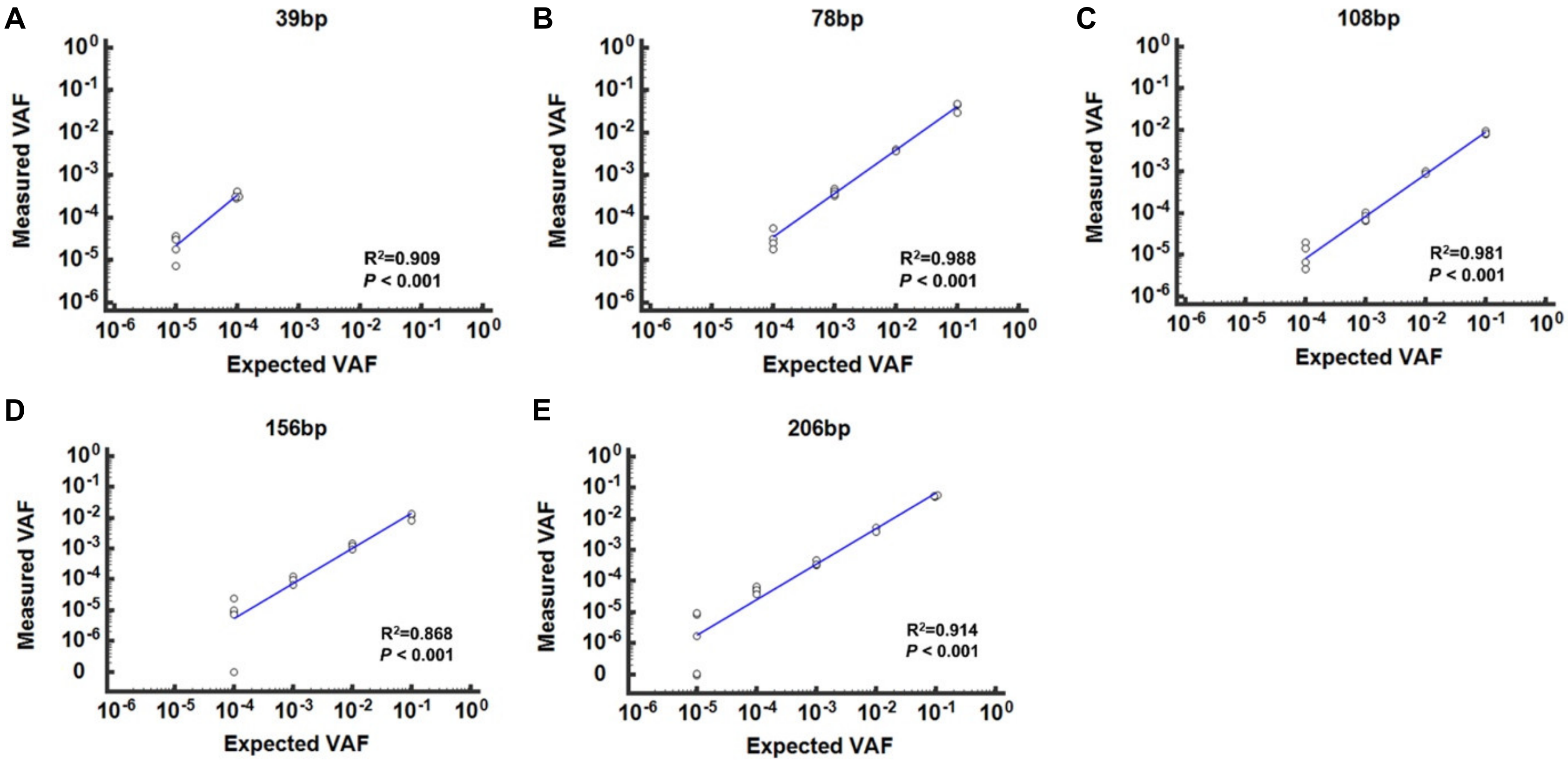

Figure S4. Linearity analyses using clinical materials.

NGS-SEED showed the highest correlation and R^2 value (A), followed by Pindel (B), Get ITD (C), and ITD seek (D) relative to FA. Red, equality line; blue, regression line.

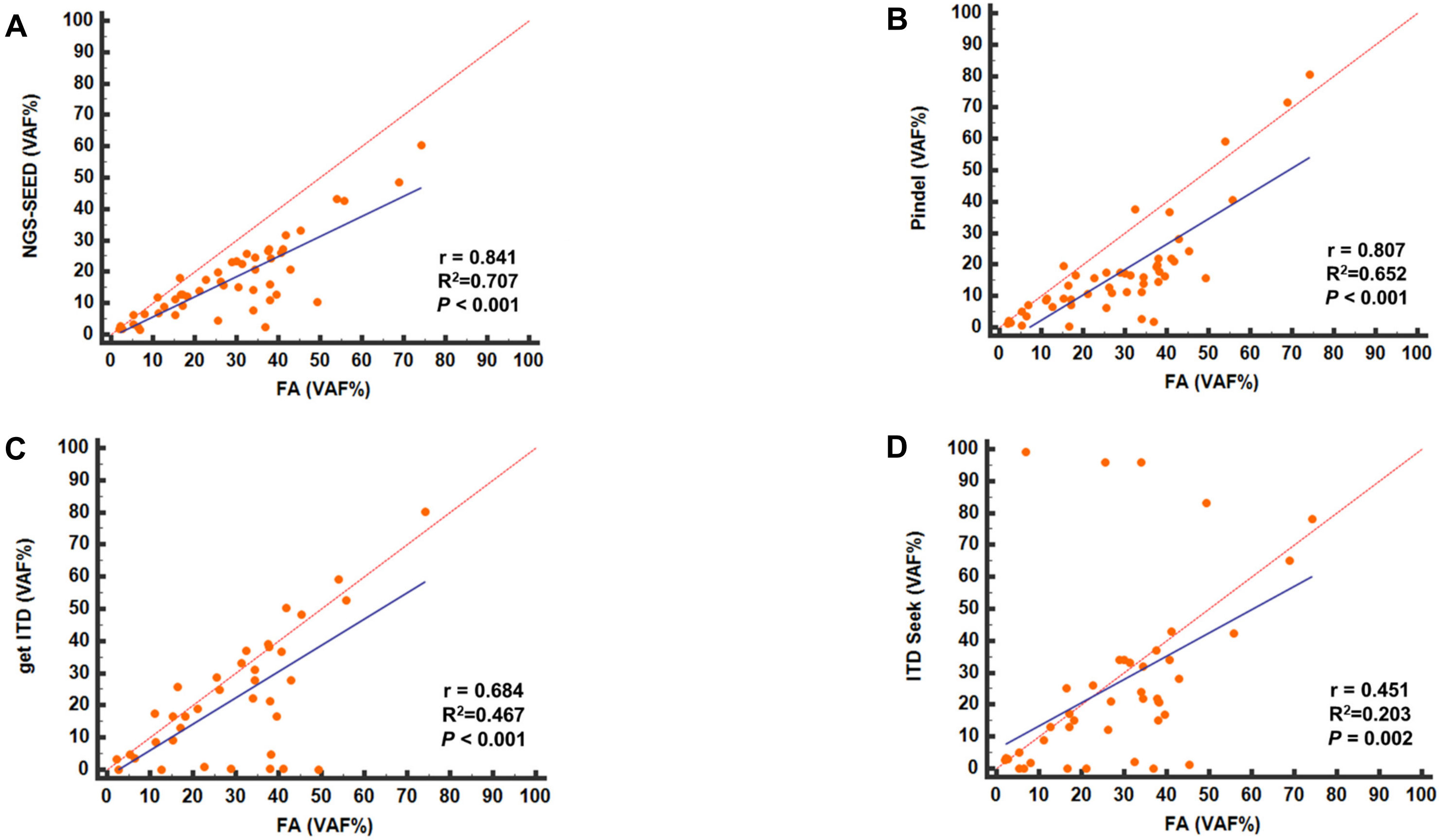

Figure S5. Regression analysis of allelic burden between FA and four bioinformatic tools.

Cumulative relapse incidence for patients with pre-HSCT MRD (A-D); post-HSCT MRD (E-F). Red, positive MRD; black, negative MRD.

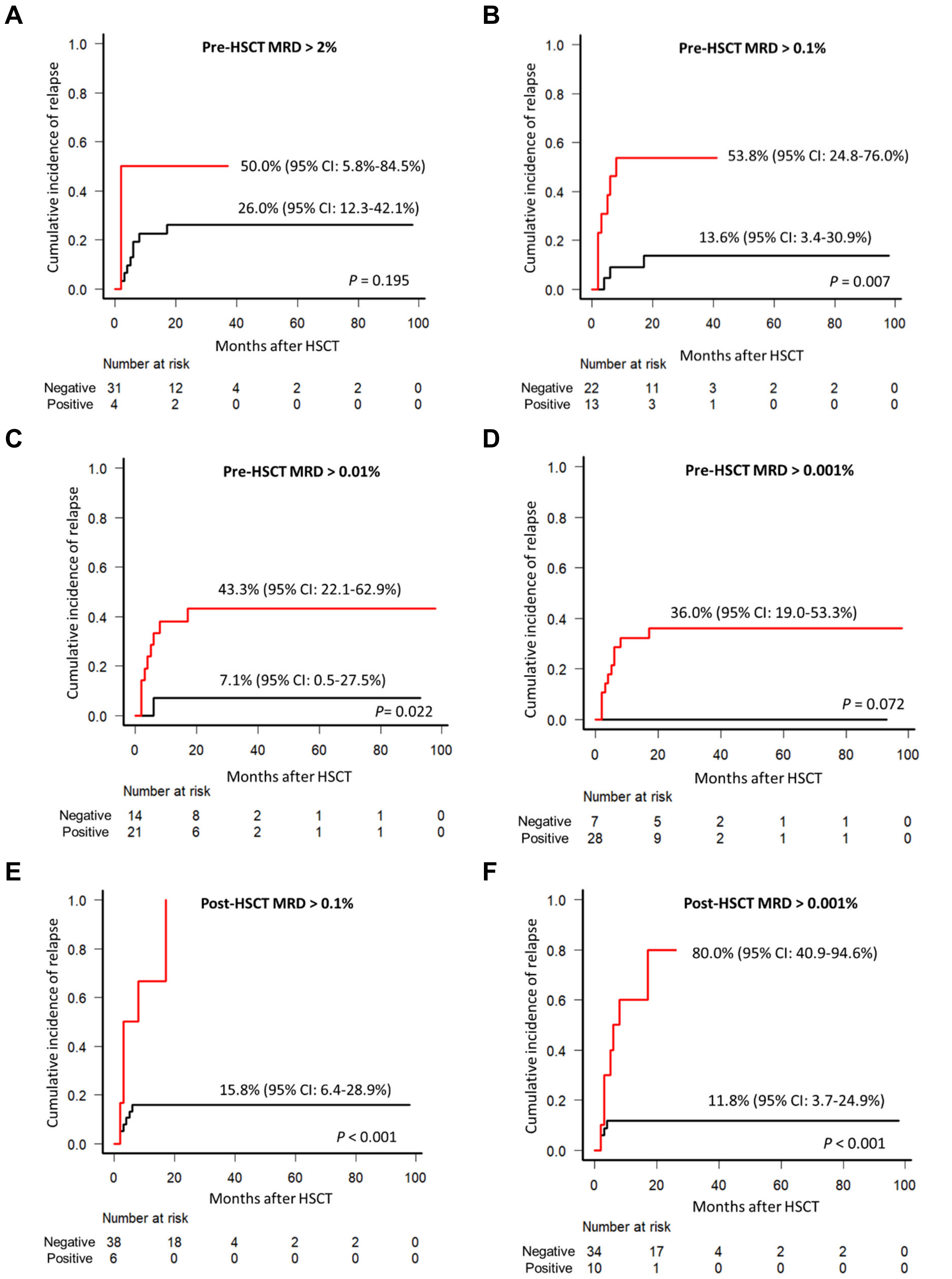

Figure S6. Relapse probability according to the *FLT3*-ITD MRD status measured by NGS-SEED.

Event free survival probability (A, B) and survival probability (C, D) for patients with post-HSCT MRD. Red, positive MRD; black, negative MRD.

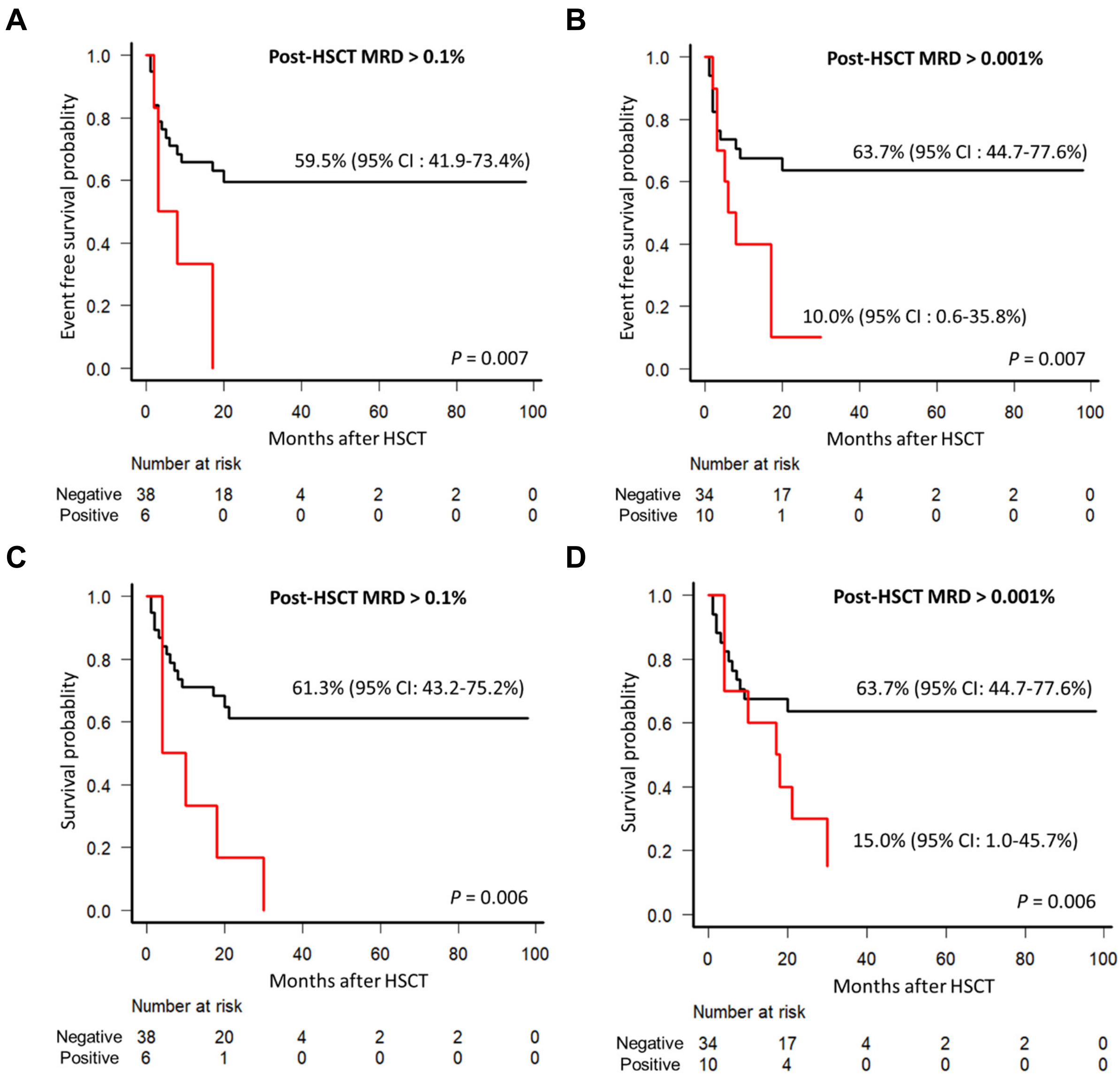

Figure S7. Survival probability according to the *FLT3*-ITD MRD status measured by NGS-SEED.
