# Supplementary material for: FLT3-ITD Measurable Residual Disease Monitoring in Acute Myeloid Leukemia Using Next-Generation Sequencing
Source: Cancers (Basel). 2022 Dec 12;14(24):6121. doi: 10.3390/cancers14246121 (PMC9776673; doi:10.3390/cancers14246121)
Supplement: Supplementary file 1 [file cancers-14-06121-s001.zip › cancers-2011631-supplementary/Cancers__Supplementary_Table R.pdf]

Table S1. Negative samples from patients with AML.

Age	Sex	Karyotype	Mutation	Total read	ITD read
65	F	46,XX[20]	*GATA2, MYC, SMC1A*	794,641	0
34	M	46,XY,r(7)(p22q22),t(12;12)(q12;q24.3),inv(16)(p13.1q22),-17,der(19)t(17;19)(q11.1;q13.4),+mar[18]/46,XY[2]	*NRAS, NF1*	497,593	0
71	M	46,XY,der(7)t(1;7)(q12;q22)[9]/46,XY[11]	*FLT3, BCOR, RUNX1, ZRSR2*	454,159	0
55	M	41,XY,-5,der(?7)del(7)(p15)inv(7)(p15q11.2),der(?10)add(10)(p15)add(10)(q24),-13,add(13)(q14),del(16)(q11.2),-17,-18,add(19)(p13.3),-21,add(21)(p11.2)[cp9]/41~42,X,-Y,+der(?1)del(1)(p22p13)del(1)(q11),del(4)(q21),-5,-7,-10,add(11)(p15),-13,add(13),-15,del(16),add(17)(p11.2),add(19),add(21)[cp10]/46,XY[1]	*TP53*	458,228	0
79	F	46,XX[10]	*NPM1, DDX41, WT1*	528,376	0
77	F	55,XX,+del(1)(q23),+2,del(5)(q13),+8,+11,+13,+19,+20,+21,+22[19]/46,XX[1]	*TP53, DDX41*	621,150	0
68	M	46,XY,der(10)t(3;10)(q13.2;p11.2)[3]/46,XY[17]	*NPM1, ASXL1, NRAS, TET2*	641,181	0
62	M	45,XY,-7[6]/46,XY[14]	*TET2*	692,681	0
60	M	45,X,-Y[3]/46,XY[27]	*RUNX1, DNMT3A*	581,072	0
58	M	46,XY[20]	*CEBPA*	347,110	0

Table S2. *FLT3*-ITDs used for dilution assay and detection.

Position	ITD size (bp)	Detection result*				ITD and SEED sequence**
		1-10%	0.1-1%	0.01-0.1%	0.001-0.01%	
28608247	39	-	-	5/5	5/5	GTTGATTTCAGAGAATATGAATATGATCTCAAATG CCTTGTTGATTTCAGAGAATATGAATATGATCTCAA ATG
28608198	78	3/3	3/3	5/5	5/5	!CTCCCATTTGAGATCATATTCATATTCTCTG GA TTGGCACATTCCATTCTTACCAAACTCTAAATTTTCTC TTGGAAACTCCCATTTGAGATCATATTCATATTCTCTG
28608222	108	3/3	3/3	5/5	5/5	CTCTAAATTTTCTCTTGGAAACTCCCATTTGAGATCATATTCATATTCTCTGAAATCAACGTAGAAGTACTC ATTATCTGAGGAGCCGGTCACCTGTACCATCTGTAG CTCTAAATTTTCTCTTGGAAACTCCCATTTGAGA TCATATTCATATTCTCTGAAATCAACGTAGAAGTACTCATTATCTGAGGAGCCGGTCACCTGTACCATCTG TAG
28608107	156	3/3	3/3	5/5	4/5	CACCTGATCCTAGTACCTTCCCTGCAAAGACAAATGGTGAGTACGTGCATTTTAAAGATTTTCCAATGGAA AAGAAATGCTGCAGAAACATTTGGCACATTCCATTCTTACCAAACTCTAAATTTTCTCTTGGAAACTCCCAT TTGAGATCATATTCACCTGATCCTAGTACCTTCCCTGCAAAGACAAATGGTGAGTACGTGCATTTTAAAGA TT!
28608102	206	3/3	3/3	5/5	3/5	AAAAGCACCTGATCCTAGTACCTTCCCTGCAAAGACAAATGGTGAGTACGTGCATTTTAAAGATTTTCCAA TGGAAAAGAAATGCTGCAGAAACATTTGGCACATTCCATTCTTACCAAACTCTAAATTTTCTCTTGGAAACT CCCATTTGAGATCATATTCATATTCTCTGAAATCAACGTAGAAGTACTCATTATCTGA TTGAAAAGCACCT GATCCTA!

*The detection results of NGS-SEED are presented as 'detected replicates / total replicates'

**The SEED sequence is indicated as colored letters (red and blue). The blue letter indicates spacer sequences. Exclamation mark indicates the presence of unrevealed sequences due to amplicon restriction.

Table S3. ITD and SEED sequences in clinical samples.

Position	ITD length	Spacer length	SEED length	ITD and SEED sequence*
28608214	36	0	14	ATGGGAGTTTCCAAGAGAAAATTTAGAGTTTGGTAA ATGGGAGTTTCCAAGAGAAAATTTAGAGTTTGGTAA
28608256	54	5	15	CCTCAGATAATGAGTACTTCTACGTTGATTTCAGAGAATATGAATATGATGGTCCCTCAGATAATGAGTACTTCT<br>ACGTTGATTTCAGAGAATATGAATATGA
28608251	21	0	18	GAGAATATGAATATGATCTCA GAGAATATGAATATGATCTCA
28608225	36	0	14	TATGATCTCAAATGGGAGTTTCCAAGAGAAAATTTA TATGATCTCAAATGGGAGTTTCCAAGAGAAAATTTA
28608237	48	2	14	CGTTGATTTCAGAGAATATGAATATGATCTCAAATGGGAGTTTCCAGGCGTTGATTTCAGAGAATATGAATATGATCTCAAATG<br>GGAGTTTCCA
28608262	21	4	14	GATTTCAGAGAATATGA GGAA GATTTCAGAGAATATGA
28608262	24	0	14	CTACGTTGATTTCAGAGAATATGA CTACGTTGATTTCAGAGAATATGA
28608262	54	1	15	GTGACCGGCTCCTCAGATAATGAGTACTTCTACGTTGATTTCAGAGAATATGAGGTGACCGGCTCCTCAGATAATGAGTACTT<br>CTACGTTGATTTCAGAGAATATGA
28608267	27	8	14	CTACGTTGATTTCAGAGAA GATGAGGG CTACGTTGATTTCAGAGAA
28608275	63	0	14	!GTGACCGGCTCCTCAGATAATGAGTACTTCTACGTTGATTCCAGCCAGCTACAGATGGTACAGGTGACCGGCTCCTCAGAT<br>AATGAGTACTTCTACGTTGATT
28608252	24	0	14	TTCAGAGAATATGAATATGATCTC TTCAGAGAATATGAATATGATCTC
28608250	27	0	14	TTTCAGAGAATATGAATATGATCTCAA TTTCAGAGAATATGAATATGATCTCAA
28608198	76	2	14	!CTCCCATTTGAGATCATATTCATATTCTCTGGATTGGCACATTCCATTCTTACCAAACTCTAAATTTTCTCTTGGAAACTCCCAT<br>TTGAGATCATATTCATATTCTCTG
28608107	156	0	14	CACCTGATCCTAGTACCTTCCCTGCAAAGACAAATGGTGAGTACGTGCATTTTAAAGATTTTCCAATGGAAAAGAAATGCTGC<br>AGAAACATTTGGCACATTCCATTCTTACCAAACTCTAAATTTTCTCTTGGAAACTCCCATTTGAGATCATATT<br>CACCTGATCCTAGTACCTTCCCTGCAAAGACAAATGGTGAGTACGTGCATTTTAAAGATT!
28608217	36	1	15	AAATGGGAGTTTCCAAGAGAAAATTTAGAGTTTGG G AAATGGGAGTTTCCAAGAGAAAATTTAGAGTTTGG
28608255	57	4	12	GCTCCTCAGATAATGAGTACTTCTACGTTGATTTCAGAGAATATGAATATGATTTCCGCTCCTCAGATAATGAGTACTTCTACGT<br>TGATTTCAGAGAATATGAATATGAT
28608220	48	0	14	ATATGAATATGATCTCAAATGGGAGTTTCCAAGAGAAAATTTAGAGTTATATGAATATGATCTCAAATGGGAGTTTCCAAGAGAA<br>AATTTAGAGTT
28608229	48	0	12	TTTTCTCTTGGAAACTCCCATTTGAGATCATATTCATATTCTCTGAAATTTTCTCTTGGAAACTCCCATTTGAGATCATATTCATA<br>TTCTCTGAAA
28608262	57	7	15	ACCGGCTCCTCAGATAATGAGTACTTCTACGTTGATTTCAGAGAATATGACAGAGGAACCGGCTCCTCAGATAATGAGTACTT<br>CTACGTTGATTTCAGAGAATATGA
28608247	39	4	14	GTTGATTTCAGAGAATATGAATATGATCTCAAATG CCTT GTTGATTTCAGAGAATATGAATATGATCTCAAATG
28608253	27	4	14	TTCAGAGAATATGAATATGATCT ATTA TTCAGAGAATATGAATATGATCT
28608221	57	3	15	TCAGAGAATATGAATATGATCTCAAATGGGAGTTTCCAAGAGAAAATTTAGAGTTTGTCAGAGAATATGAATATGATCTCAAAT<br>GGGAGTTTCCAAGAGAAAATTTAGAGT
28608299	84	0	15	TAATGAGTACTTCTACGTTGATTTCAGAGAATATGAATATGATCTCAAATGGGAGTTTCCAAGAGAAAATTTAGAGTTTGG<br>GCTTAATGAGTACTTCTACGTTGATTTCAGAGAATATGAATATGATCTCAAAT!
28608313	45	0	14	GACCGGCTCCTCAGATAATGAGTACTTCTACGTTGATTTCAGAGAGACCGGCTCCTCAGATAATGAGTACTTCTACGTTGATT<br>TCAGAGA

28608282	36	3	15	!GTGACCGGCTCCTCAGATAATGAGTACTTCTAC CAG GTGACCGGCTCCTCAGATAATGAGTACTTCTAC
28608199	60	0	14	CTCAAATGGGAGTTTCCAAGAGAAAATTTAGAGTTTGGTAAGAATGGAATGTGCCAGATGCTCAAATGGGAGTTTCCAAGAGAAAATTTAG!
28608262	27	0	14	CTTCTACGTTGATTTCAGAGAATATGA CTTCTACGTTGATTTCAGAGAATATGA
28608268	18	2	14	ACGTTGATTTCAGAGA CC ACGTTGATTTCAGAGA
28608215	69	0	14	ACGTTGATTTCAGAGAATATGAATATGATCTCAAATGGGAGTTTCCAAGAGAAAATTTAGAGTTTGGTAACGTTGATTTCAGAGAATATGAATATGATCTCAAATGGGAGTTTCCAAGAG!
28608201	75	0	14	GAGAATATGAATATGATCTCAAATGGGAGTTTCCAAGAGAAAATTTAGAGTTTGGTAAGAATGGAATGTGCGCCGGAGAATATGAATATGATCTCAAATGGGAGTTT!
28608239	72	2	14	GGCTCCTCAGATAATGAGTACTTCTACGTTGATTTCAGAGAATATGAATATGATCTCAAATGGGAGTTTCAAGGCTCCTCAGATAATGAGTACTTCTACGTTGATTTCAGAGAATATGAATATGATCTCAAATGGGAGTTTC
28608255	21	0	14	TTCAGAGAATATGAATATGAT TTCAGAGAATATGAATATGAT
28608228	54	0	14	GTTGATTTCAGAGAATATGAATATGATCTCAAATGGGAGTTTCCAAGAGAAAATGTTGATTTCAGAGAATATGAATATGATCTCAAATGGGAGTTTCCAAGAGAAAAT
28608242	39	1	15	TGATTTCAGAGAATATGAATATGATCTCAAATGGGAGTCTGATTTCAGAGAATATGAATATGATCTCAAATGGGAGT
28608271	36	0	14	CTCCTCAGATAATGAGTACTTCTACGTTGATTTCAG CTCCTCAGATAATGAGTACTTCTACGTTGATTTCAG
28608268	24	4	16	TTCTACGTTGATTTCAGAGA GGGC TTCTACGTTGATTTCAGAGA
28608249	66	5	15	CGGCTCCTCAGATAATGAGTACTTCTACGTTGATTTCAGAGAATATGAATATGATCTCAAAAGGAACGGCTCCTCAGATAATGAGTACTTCTACGTTGATTTCAGAGAATATGAATATGATCTCAAA
28608244	21	0	14	TGAATATGATCTCAAATGGGA TGAATATGATCTCAAATGGGA
28608285	39	13	17	CCGGCTCCTCAGATAATGAGTACTTC CAAATGAGTATTT CCGGCTCCTCAGATAATGAGTACTTC
28608267	36	3	15	GATAATGAGTACTTCTACGTTGATTTCAGAGAA TTT GATAATGAGTACTTCTACGTTGATTTCAGAGAA
28608220	63	0	14	ATTTCAGAGAATATGAATATGATCTCAAATGGGAGTTTCCAAGAGAAAATTTAGAGTTAGGAGATTTCAGAGAATATGAATATGATCTCAAATGGGAGTTTCCAAGAGAAAATT!
28608261	66	12	18	!GTGACCGGCTCCTCAGATAATGAGTACTTCTACGTTGATTTCAGAGAATATGAACAGATGGTACAGGTGACCGGCTCCTCAGATAATGAGTACTTCTACGTTGATTTCAGAGAATATGAA
28608271	42	2	14	CCGGCTCCTCAGATAATGAGTACTTCTACGTTGATTTCAGGGCCGGCTCCTCAGATAATGAGTACTTCTACGTTGATTTCAG
28608244	18	0	14	ATATGATCTCAAATGGGA ATATGATCTCAAATGGGA
28608268	30	0	14	TAATGAGTACTTCTACGTTGATTTCAGAGA TAATGAGTACTTCTACGTTGATTTCAGAGA
28608215	51	4	14	ATATGATCTCAAATGGGAGTTTCCAAGAGAAAATTTAGAGTTTGGTAACGGATATGATCTCAAATGGGAGTTTCCAAGAGAAAATTTAGAGTTTGGTA!
28608255	84	0	13	!GTGACCGGCTCCTCAGATAATGAGTACTTCTACGTTGATTTCAGAGAATATGAATATGATGAAAGCCAGCTACAGATGGTACAGGTGACCGGCTCCTCAGATAATGAGTACTTCTACGTTGATTTCAGAGAATATGAATATGAT
28608262	54	1	14	TCATATTCTCTGAAATCAACGTAGAAGTACTCATTATCTGAGGAGCCGGTCACCTCATATTCTCTGAAATCAACGTAGAAGTACTCATTATCTGAGGAGCCGGTCAC
28608271	48	4	14	CTGAAATCAACGTAGAAGTACTCATTATCTGAGGAGCCGGTCACAGAGCTGAAATCAACGTAGAAGTACTCATTATCTGAGGAGCCGGTCAC
28608217	75	5	14	!ACTCCCATTTGAGATCATATTCATATTCTCTGAAATCAACGTAGAAGTCGCCCCCAAACTCTAAATTTTCTCTTGGAAACTCCCATTTGAGATCATATTCATATTCTCTGAAATCAACGTAGAAGT
28608268	51	6	14	TCTCTGAAATCAACGTAGAAGTACTCATTATCTGAGGAGCCGGTCTCATAATCTCTGAAATCAACGTAGAAGTACTCATTATCTGAGGAGCCGGTC
28608222	109	0	14	CTCTAAATTTTCTCTTGGAAACTCCCATTTGAGATCATATTCATATTCTCTGAAATCAACGTAGAAGTACTCATTATCTGAGGAG

				CCGGTCACCTGTACCATCTGTAGCTCTAAATTTTCTCTTGGAAACTCCCATTTGAGATCATATTCATATTCTCTGAAATCAACG<br>TAGAAGTACTCATTATCTGAGGAGCCGGTCACCTGTACCATCTGTAG
28608102	205	3	15	AAAAGCACCTGATCCTAGTACCTTCCCTGCAAAGACAAATGGTGAGTACGTGCATTTTAAAGATTTTCCAATGGAAAAGAAAT<br>GCTGCAGAAACATTTGGCACATTCCATTCTTACCAAACTCTAAATTTTCTCTTGGAAACTCCCATTTGAGATCATATTCATATTC<br>TCTGAAATCAACGTAGAAGTACTCATTATCTGA TTG AAAAGCACCTGATCCTA!

*The SEED sequence is shown in red and blue; spacer sequences are in blue. Exclamation marks indicate unavailable sequences due to amplicon restriction.

Table S4. Detection capacities of bioinformatic tools.

Locus				Length					Allele burden (%)				
SEED	Pindel	ITDseek	getITD	FA	SEED	Pindel	ITDseek	getITD	FA	SEED	Pindel	ITDseek	getITD
28608268	28608268	28608268	28608269	18	18	18	18	18	0.742	0.6046	0.8038	0.78	0.8021
28608244	28608244	28608244	28608244	18	18	18	18	18	0.262	0.1682	0.1277	0.12	0.2497
28608262	28608262	28608262	28608262	20	21	21	21	21	0.054	0.0332	0.0492	0.05	0.0467
28608255	28608255	28608255	28608255	20	21	21	21	21	0.377	0.2698	0.1952	0.22	0.3813
28608244	28608244	28608244	28608244	20	21	21	21	21	0.112	0.1185	0.0879	0.09	0.1743
28608251	28608251	28608251	28608252	21	21	21	21	21	0.382	0.2421	0.1766	0.2077	0.0483
28608268	28608268	28608268	28608268	23	24	24	24	24	0.407	0.2599	0.3678	0.34	0.3661
28608262	28608262	28608262	28608262	24	24	24	24	24	0.182	0.1226	0.1655	0.15	0.1651
28608253	28608253	28608253	28608253	26	27	27	27	27	0.344	0.207	0.1382	0.22	0.2787
28608262	28608262	28608262	28608262	26	27	27	27	27	0.064	0.0231	0.0357	0.0002	0.0364
28608267	28608267	28608267	ND	27	27	27	27	ND	0.688	0.4848	0.7168	0.65	ND
28608268	28608268	ND	28608268	29	30	30	ND	30	0.54	0.4308	0.5918	ND	0.5906
28608267	28608266	ND	28608267	34	36	36	ND	36	0.153	0.0621	0.0924	ND	0.0928
28608214	28608214	ND	28608214	35	36	36	ND	36	0.153	0.1126	0.1944	ND	0.165
28608217	28608217	28608217	28608217	35	36	36	36	36	0.212	0.1384	0.1077	0.0002	0.189
28608282	28608282	28608282	ND	35	36	36	36	ND	0.34	0.0755	0.1125	0.96	ND
28608225	28608225	28608225	28608226	36	36	36	36	36	0.165	0.1807	0.1326	0.25	0.2575
28608271	28608271	28608271	28608271	36	36	36	37	36	0.325	0.2568	0.3744	0.02	0.3694
28608247	28608247	28608247	28608247	37	39	39	39	39	0.453	0.3323	0.2433	0.0113	0.4827
28608242	28608242	28608242	28608242	38	39	39	38	39	0.376	0.269	0.1961	0.37	0.3904
28608285	28608285	ND	28608285	39	39	39	ND	39	0.122	0.0764	0.106	ND	0.1025
28608271	28608271	28608271	28608271	41	42	42	40	42	0.428	0.2084	0.2815	0.28	0.2777
28608313	28608313	28608313	28608313	45	45	45	45	45	0.379	0.1588	0.2177	0.21	0.2119
28608237	28608237	28608236	28608237	46	48	48	48	48	0.023	0.0238	0.0174	0.03	0.0335
28608220	28608220	28608219	ND	46	48	48	50	ND	0.17	0.1241	0.0876	0.1727	ND
28608271	28608271	28608271	28608271	46	48	48	44	48	0.38	0.1082	0.1448	0.15	0.003
28608229	28608226	ND	28608226	47	48	48	ND	48	0.417	0.3153	0.2099	ND	0.5036
28608215	28608217	28608213	ND	49	51	51	50	ND	0.3	0.2346	0.1718	0.34	ND
28608268	28608268	28608262	28608268	49	51	51	51	51	0.396	0.1275	0.1619	0.17	0.1658
28608256	28608255	ND	28608256	51	54	54	ND	54	0.255	0.1969	0.1746	ND	0.288
28608262	28608262	28608262	28608262	51	54	54	55	51	0.493	0.1049	0.1579	0.83	0.0012
28608228	28608228	28608227	28608228	52	54	54	56	54	0.314	0.2259	0.1644	0.33	0.3304
28608262	28608262	ND	28608262	55	57	57	ND	57	0.114	0.0667	0.0884	ND	0.0853
28608221	28608223	28608218	28608219	55	57	57	59	56	0.41	0.2721	0.2179	0.4296	0.0022
28608255	28608255	28608255	28608255	56	57	57	53	57	0.345	0.247	0.1596	0.32	0.3117
28608199	28608226*	28608199	28608197	58	60	60	58	59	0.558	0.4255	0.4068	0.4219	0.526

28608220	28608229*	28608219	ND	60	63	63	61	ND	0.021	0.0198	0.0146	0.03	ND
28608275	28608275	28608275	ND	62	63	63	61	ND	0.068	0.0147	0.072	**0.99**	ND
28608249	28608249	ND	ND	63	66	66	ND	ND	0.305	0.1518	0.1123	ND	ND
28608261	28608261	28608261	ND	65	66	66	67	ND	0.255	0.0432	0.0627	**0.96**	ND
28608215	28608235*	28608213	28608215	66	69	69	72	68	0.027	0.0178	0.0138	0.03	0.0002
28608239	28608239	28608239	ND	69	72	72	70	ND	0.269	0.1581	0.1092	0.21	ND
28608201	28608242*	28608200	28608202	73	75	75	73	74	0.227	0.1737	0.158	0.26	0.0102
28608198	28608198	28608197	28608198	75	76	78	78	78	0.34	0.1433	0.028	0.24	0.223
28608217	28608244*	28608216	28608218	76	75	78	75	77	0.289	0.2318	0.1747	0.34	0.0023
28608299	28608250*	28608299	28608301	81	84	84	83	83	0.126	0.0893	0.0646	0.13	0.0009
28608255	28608255	28608255	ND	81	84	84	86	ND	0.369	0.0238	0.0163	0.0005	ND
28608222	ND	28608222	ND	106	109	ND	112	ND	0.079	0.0664	ND	0.019	ND
28608107	28608107	28608106	ND	153	156	156	160	ND	0.054	0.0629	0.007	0.0013	ND
28608102	28608102	28608101	ND	206	205	204	203	ND	0.167	0.1316	0.002	0.0002	ND

Gray shading indicates relative VAF levels. Significantly discordant results are highlighted in yellow.

Table S5. Predicting performance of NGS-SEED MRD for clinical outcomes according to various cut-offs at each time point.

Clinical outcome	Time point	Cut-off	Sensitivity	Specificity	Positive predictive value	Negative predictive value	Area Under the Curve	P value
Relapse	Pre-HSCT	**0.1%***	70.0%	76.0%	53.9%	86.4%	0.730	0.009
		0.01%	90.0%	52.0%	42.9%	92.9%	0.710	0.003
		0.001%	100%	28.0%	35.7%	100%	0.640	0.002
	Post-HSCT	0.1%	44.4%	100%	100%	83.1%	0.722	0.288
		**0.001%***	55.5%	93.9%	76.9%	85.2%	0.747	<0.001
Event free survival	Pre-HSCT	0.1%	47.1%	72.2%	61.5%	59.1%	0.596	0.244
		**0.01%**	76.5%	55.6%	61.9%	71.4%	0.660	0.046
		0.001%	88.2%	27.8%	53.6%	71.4%	0.580	0.236
	Post-HSCT	0.1%	78.6%	52.6%	55.0%	76.9%	0.656	0.016
		**0.001%***	82.1%	50.0%	54.8%	79.2%	0.661	0.002
Overall survival	Pre-HSCT	0.1%	43.8%	68.4%	53.8%	59.1%	0.561	0.470
		**0.01%**	75.0%	52.6%	57.1%	71.4%	0.638	0.089
		0.001%	87.5%	26.3%	50.0%	71.4%	0.569	0.304
	Post-HSCT	**0.1%***	80.8%	53.7%	52.5%	81.5%	0.672	0.010
		0.001%	84.6%	48.8%	51.2%	83.3%	0.667	0.002

MRD cut-offs with the highest area under the curve are highlighted in bold; * MRD factors that included in the subsequent multivariate analysis.
